# Global patterns of water storage in the rooting zones of vegetation

**DOI:** 10.1038/s41561-023-01125-2

**Published:** 2023-02-09

**Authors:** Benjamin D. Stocker, Shersingh Joseph Tumber-Dávila, Alexandra G. Konings, Martha C. Anderson, Christopher Hain, Robert B. Jackson

**Affiliations:** 1 Department of Earth System Science, Stanford University, Stanford, CA USA; 2 Department of Environmental Systems Science, ETH, Zürich, Switzerland; 3 Swiss Federal Institute for Forest, Snow and Landscape Research WSL, Birmensdorf, Switzerland; 4 Institute of Geography, University of Bern, Bern, Switzerland; 5 Oeschger Centre for Climate Change Research, University of Bern, Bern, Switzerland; 6 Harvard Forest, Harvard University, Petersham, MA USA; 7 USDA-ARS Hydrology and Remote Sensing Laboratory, Beltsville, MD USA; 8 NASA Marshall Space Flight Center, Redstone Arsenal, AL USA; 9 Woods Institute for the Environment, Stanford University, Stanford, CA USA; 10 Precourt Institute for Energy, Stanford University, Stanford, CA USA

**Keywords:** Ecosystem ecology, Hydrology, Climate-change ecology

## Abstract

The rooting-zone water-storage capacity—the amount of water accessible to plants—controls the sensitivity of land–atmosphere exchange of water and carbon during dry periods. How the rooting-zone water-storage capacity varies spatially is largely unknown and not directly observable. Here we estimate rooting-zone water-storage capacity globally from the relationship between remotely sensed vegetation activity, measured by combining evapotranspiration, sun-induced fluorescence and radiation estimates, and the cumulative water deficit calculated from daily time series of precipitation and evapotranspiration. Our findings indicate plant-available water stores that exceed the storage capacity of 2-m-deep soils across 37% of Earth’s vegetated surface. We find that biome-level variations of rooting-zone water-storage capacities correlate with observed rooting-zone depth distributions and reflect the influence of hydroclimate, as measured by the magnitude of annual cumulative water-deficit extremes. Smaller-scale variations are linked to topography and land use. Our findings document large spatial variations in the effective root-zone water-storage capacity and illustrate a tight link among the climatology of water deficits, rooting depth of vegetation and its sensitivity to water stress.

## Abstract (web summary)

Global estimations of the water-storage capacity in the rooting zone from satellite data reveal plant access to deep water across a third of Earth’s vegetated surface.

## Main

To sustain activity during dry periods and resist impacts of droughts, plants rely on water stored below the surface. The larger the rooting-zone water-storage capacity (*S*_0_), the longer plants can withstand soil moisture limitation^1^. *S*_0_ is therefore a key factor determining drought impacts, land–atmosphere exchanges and run-off regimes, particularly in climates with a seasonal asynchrony in radiation and precipitation (*P*)^2–4^. In models, *S*_0_ is commonly conceived as a function of the soil texture and the plants’ rooting depth (*z*_r_), limited to the depth of the soil^3,5^. Recent research has revealed a substantial component of *S*_0_ and contributions to evapotranspiration (ET) by water stored beneath the soil, in weathered and fractured bedrock and groundwater^6–11^. Plant access to such deep moisture plays an important role in controlling near-surface climate^12–14^, run-off regimes^4^, global patterns of vegetation cover^15^ and mitigating impacts of droughts^16^.

However, *S*_0_ is impossible to observe directly across large scales, and its spatial variations are poorly understood^17^. Global compilations of local plant *z*_r_ measurements^18,19^ yield information related to *S*_0_ but have resolved this observational challenge only partly because of their limited size and large documented variations in *z*_r_ across multiple scales^7,18,18–21^. Empirical approaches for estimating the global *z*_r_ distribution have made use of relationships between in situ observations and climatic factors^22^. Modelling approaches for predicting *z*_r_ have conceived their spatial variations as the result of optimal adaptation to the prevailing hydroclimate^23–25^ or as being adapted to just buffer water demand to sustain ET during dry periods^2,26^. Such mass-balance approaches make use of the maximum cumulative water deficit (CWD) during dry periods as an indication of the effective *S*_0_. An additional hypothesis posits that it would not be beneficial for plants to root even deeper and thus size their *S*_0_ even larger^26^. However, a link among the magnitude of CWD extremes, the sensitivity of vegetation activity to an increasing CWD and local *z*_r_ observations remains to be shown, and the prevalence of plant access to water stored at depth (here taken as >2 m) across the globe remains to be quantified.

Despite its crucial role in controlling water and carbon fluxes and the scarcity of observations, virtually all models simulating water and carbon exchange between the land surface and the atmosphere rely on a specification of *S*_0_ either directly as the depth of a ‘water bucket’ or indirectly through prescribed *z*_*r*_ and soil texture across the profile. Typically, water stored at depth and along the entire critical zone (including weathered bedrock) is not fully represented in models^8,9^, and the evident plasticity of *z*_r_ and variations of *S*_0_ within plant types and along climatic and topographic gradients are often ignored. Implications of this simplification may be substantial for the simulation of land–atmosphere coupling and drought impacts^8,12,13^.

In this Article, we present a method for diagnosing *S*_0_ from the relationship between vegetation activity and CWD. By fusing multiple time series of Earth observation data streams with global coverage, we estimate the global distribution of *S*_0_ at a resolution of 0.05° (~5 km). Using a mass-balance approach^2,26^ and field observations of *z*_r_ from a globally distributed dataset, we then show that the sensitivity of vegetation to water stress across the globe is strongly related to the magnitude of CWD extremes and reflects the rooting depth of plants.

## Estimating *S*_0_ from Earth observations

We started by estimating *S*_0_ as the CWD at which vegetation ‘activity’ ceases. Our approach accounts for the constraint of the rooting-zone water availability on ET and photosynthesis and relates *S*_0_ to the sensitivity of vegetation activity to water stress. The parallel information of ET, *P* and the modelled snow mass balance enables a quantification of CWD over time. Vegetation activity was estimated from two alternative observations: from the evaporative fraction (EF, defined as ET divided by net radiation) and from sun-induced fluorescence (SIF, normalized by incident short-wave radiation (Methods)).

**Fig. 1:**
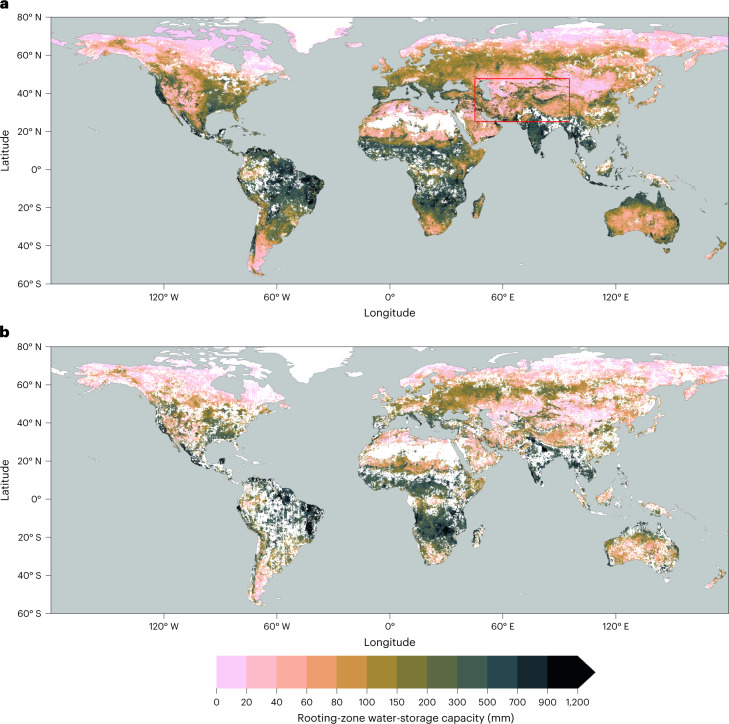
Rooting-zone water-storage capacity from vegetation activity. **a**,**b**, Rooting-zone water-storage capacity estimated from *S*_dEF_ (**a**) and *S*_dSIF_ (**b**) to the CWD. The red box in **a** shows the outline of the magnified map provided in Fig. 2. Data shown are aggregated to 0.1° resolution. Blank cells (white) mark areas where all underlying cells at the original 0.05° resolution did not exhibit a significant and single, linearly declining relationship with increasing CWD.

Figure 1 reveals large global variations in *S*_0_. Estimates based on EF and SIF correlate closely and agree in magnitude (*R*^2^ = 0.78; Supplementary Fig. 1). The lowest sensitivity of vegetation activity to an increasing CWD, and thus the largest apparent *S*_0_, is found in regions with a strong seasonality in radiation and water availability and substantial vegetation cover—particularly in monsoonal climates. By contrast, the lowest *S*_0_ values appear not only in regions where seasonal water deficits are limited due to short inter-storm duration (for example, western Amazon and Congo basin) and/or low levels of potential ET (for example, high latitudes), but also in deserts and arid grasslands. This probably reflects the limited water storage accumulating during rain events from which vegetation can draw during dry periods. In these regions, a rapid decline of ET and SIF with an increasing CWD is related to vegetation cover dynamics, governed by greening after rain pulses and browning during dry periods^27^.

**Fig. 2:**
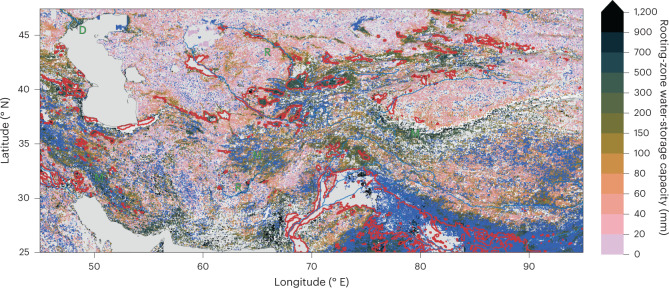
Rooting-zone water-storage capacity in Central Asia, estimated from *S*_dEF_. Blue areas (flattening) show grid cells where a significant reduction in the slope in EF versus CWD was identified beyond a certain threshold. *S*_dEF_ values are not calculated for grid cells classified as flattening. Red lines outline major irrigated areas, where the irrigated land area fraction is above 30%^41^. Information about irrigated areas was used only for mapping here, but is not used for other parts of the analysis. Blank grid cells (white) indicate areas with a sustained imbalance of ET being greater than *P*. Green letters indicate locations of mountains (M), rivers (R) and delta (D), referred to in the main text. Additional regional maps are provided by Extended Data Figs. 1–3.

**Extended Data Fig. 1:**
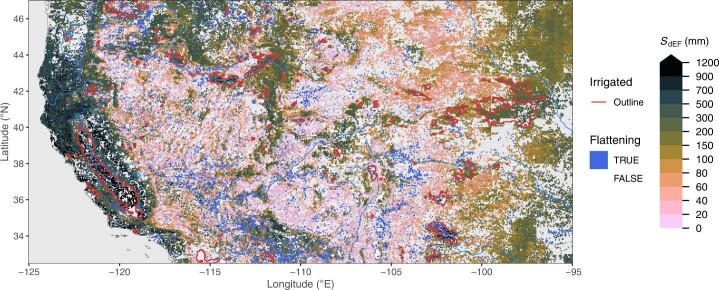
Rooting zone water storage capacity in the Western USA. Estimated from the evaporative fraction (S_dEF_). Blue areas (‘flattening’) show grid cells where a significant reduction in the slope in EF vs. CWD was identified beyond a certain threshold. Red lines show outlines of major irrigated areas, that is, where the irrigated land area fraction is above 30%^39^. Blank grid cells indicate areas with a sustained imbalance of ET being greater than *P*.

**Extended Data Fig. 2:**
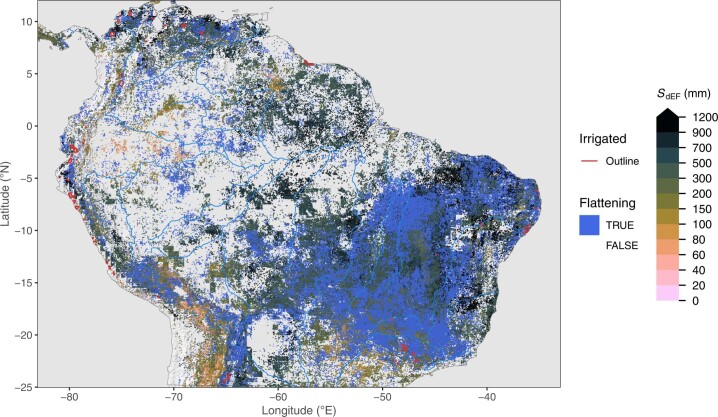
Rooting zone water storage capacity in the Amazon region. Estimated from the evaporative fraction (S_dEF_). Blue areas (‘flattening’) show grid cells where a significant reduction in the slope in EF vs. CWD was identified beyond a certain threshold. Red lines show outlines of major irrigated areas, that is, where the irrigated land area fraction is above 30%^39^. Blank grid cells indicate areas with a sustained imbalance of ET being greater than *P*.

**Extended Data Fig. 3:**
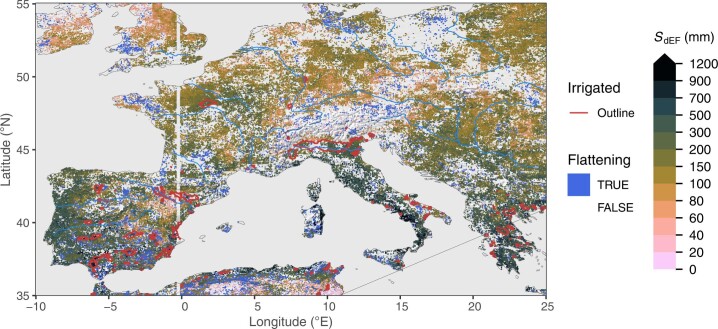
Rooting zone water storage capacity in Europe. Estimated from the evaporative fraction (S_dEF_). Blue areas (‘flattening’) show grid cells where a significant reduction in the slope in EF vs. CWD was identified beyond a certain threshold. Red lines show outlines of major irrigated areas, that is, where the irrigated land area fraction is above 30%^39^. Blank grid cells indicate areas with a sustained imbalance of ET being greater than *P*.

Clear patterns emerge also at smaller scales (Fig. 2 and Extended Data Figs. 1–3). The sensitivity of SIF (*S*_dSIF_) and sensitivity of the EF (*S*_dEF_) consistently (Supplementary Fig. 1) reveal how the sensitivity of photosynthesis and transpiration to drought stress varies across different topographical settings, indicating generally larger *S*_0_ in mountain regions (’M’ in Fig. 2) and along rivers (’R’) and deltas (’D’). We note, however, that ET estimates from the product used here (ALEXI^28,29^) may be biased high over mountainous terrain where low incident net radiation and surface temperatures are caused not by high EFs but rather by topography effects and local shading. The maps of *S*_dSIF_ and *S*_dEF_ also bear strong imprints of human land use. Major irrigated cropland areas are congruent with some of the highest apparent *S*_0_ values. In these areas, our analysis yields particularly high CWD values and a low sensitivity of SIF and EF to CWD, without using information about the location and magnitude of irrigation. Other major irrigated areas appear as blank cells in Fig. 2 because the algorithm used to calculate CWD (Methods) fails due to a long-term imbalance between *P* and ET and a ‘runaway CWD’. This indicates sustained overuse of water resources, caused by lateral water redistribution at scales beyond ~5 km via streamflow diversion or groundwater flow and extraction (or bias in *P* and ET estimates).

Regressing vegetation activity against CWD also identifies locations where a decoupling of the two variables appears, that is, where the sensitivity of EF or SIF significantly decreases beyond a certain CWD threshold (‘flattening’ in Fig. 2; Methods). Such areas are particularly common in the vicinity of mountain regions, in areas with irrigated croplands and in savannahs (Supplementary Fig. 2). Related mechanisms may be at play. A flattening of the EF (SIF) versus CWD relationship is probably due to different portions of the vegetation having access to distinct water resources and respective storage capacities. In areas with large topographic gradients, this may be due to within-grid-cell heterogeneity in plant access to the saturated zone. Although relevant for land–atmosphere coupling^12^, land surface models typically do not account for such effects. This has potential implications for simulations of ET during prolonged dry periods in these regions. In savannahs, a shift in ET contributions from grasses and trees and a related shift in transpiration occurs as grasses, which are often more shallow rooted than trees^30^, senesce. In irrigated cropland areas, the flattening probably reflects land-use heterogeneity within ~5 km grid cells and the persistent water access on irrigated fields while EF and SIF are reduced more rapidly in surrounding vegetation.

**Extended Data Fig. 4:**
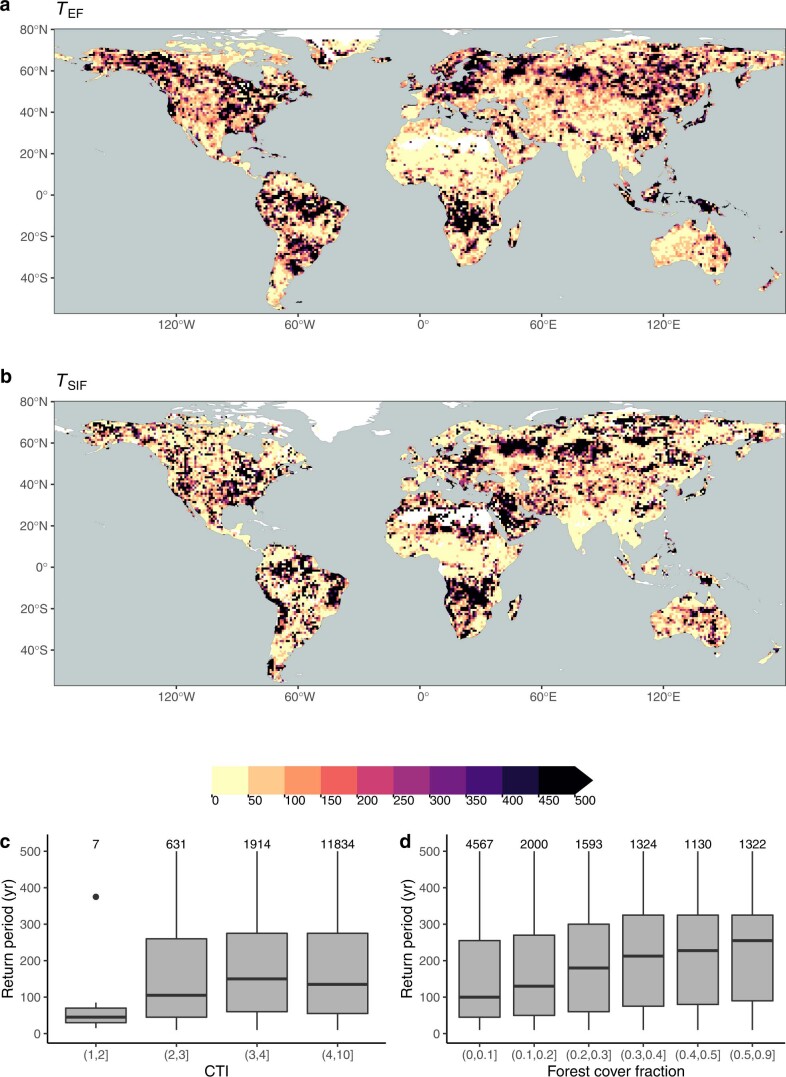
Return periods diagnosed from the evaporative fraction and sun-induced fluorescence. Return periods *T* (yr), diagnosed from EF (a) and SIF (b). To diagnose *T*, a range of alternative values of *T* are screened and the corresponding range of values *S*_CWDX*T*_ are compared to *S*_dEF_ *S*_dSIF_) within 1^∘^ grid cells (resolution of maps shown here). The best matching *T* was retained for each gridcell, yielding a global distribution of *T*_EF_ (*T*_SIF_). The bottom panel shows the distribution of diagnosed return periods *T* (mean of *T*_EF_ and *T*_SIF_) within bins of the Compound Topography Index^60^ (c) and forest cover fractions (MOD44B^62^) (d). Boxes represent the interquartile ranges of binned values (*Q*_25_, *Q*_75_), and whiskers cover *Q*_25_ − 1.5(*Q*_75_ − *Q*_25_) to *Q*_75_ + 1.5(*Q*_75_ − *Q*_25_). Numbers of data points per bin are given above boxes.

What controls spatial variations in *S*_0_ and *z*_r_ and the sensitivity of vegetation activity to water stress? Following ref. ^2^, we hypothesized that annual CWD maxima reflect the total amount of plant-accessible water. That is, *z*_r_ and *S*_0_ are sized to just maintain transpiration and photosynthesis under extreme water deficits, commonly experienced over the course of a plant’s lifetime (recurring with a return period of *T* yr). Hence, a correlation between the magnitude of CWD extremes and the sensitivity of vegetation activity to an increasing CWD should emerge. For estimating CWD extremes, we started by using *T* = 80 yr and assessed other choices as described in Supplementary Text 1 (also see Extended Data Fig. 4).

**Fig. 3:**
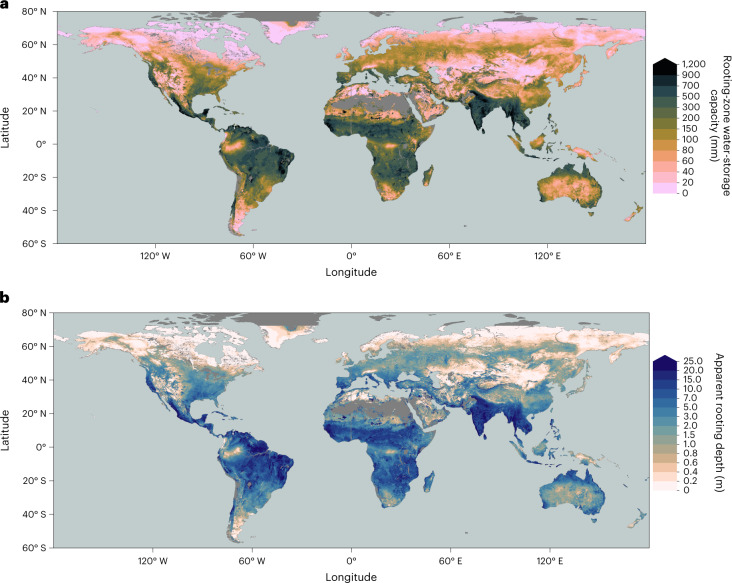
Rooting-zone water-storage capacity and apparent rooting depth from the water mass balance. **a**,**b**, Spatial variations of the rooting-zone water-storage capacity, estimated by *S*_CWDX80_ (**a**) and the apparent rooting depth *z*_CWDX80_ (**b**). Values are remapped to 0.1° resolution. Blank grid cells (grey) are either permanent inland water bodies and ocean or locations with long-term accumulation of water deficits. Values are removed in grid cells where more than 99% is non-vegetation surface according to MODIS Landcover^42^.

**Extended Data Fig. 5:**
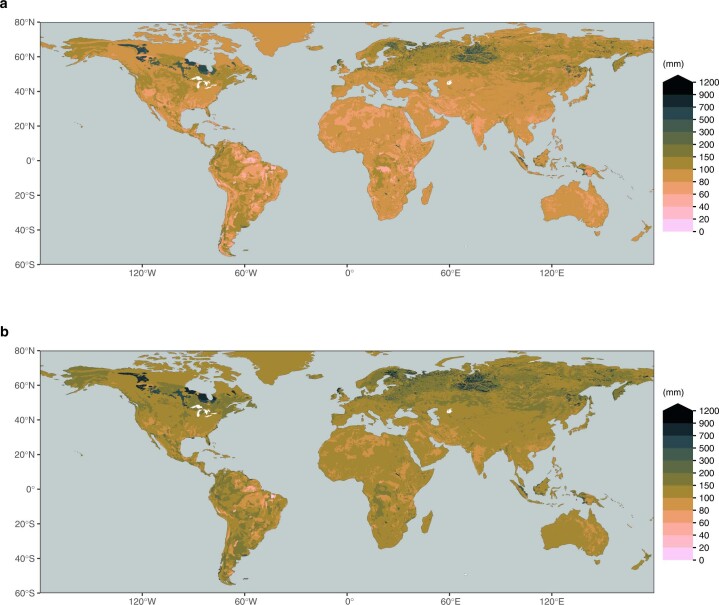
Integrated soil water holding capacity in the soil. Integrated soil water holding capacity across the top 1 m (a) and the top 2 m (b). Values are calculated based on soil texture information from a gridded version^30^ of the Harmonized World Soil Database^29^ and pedo-transfer functions based on ref. ^31^. HWSD provides information for a top layer (0-30 cm depth) and a bottom layer (30-100 cm depth). For the top 2 m shown in (b), we assumed values from the bottom layer for 100-200 cm depth.

**Extended Data Fig. 6:**
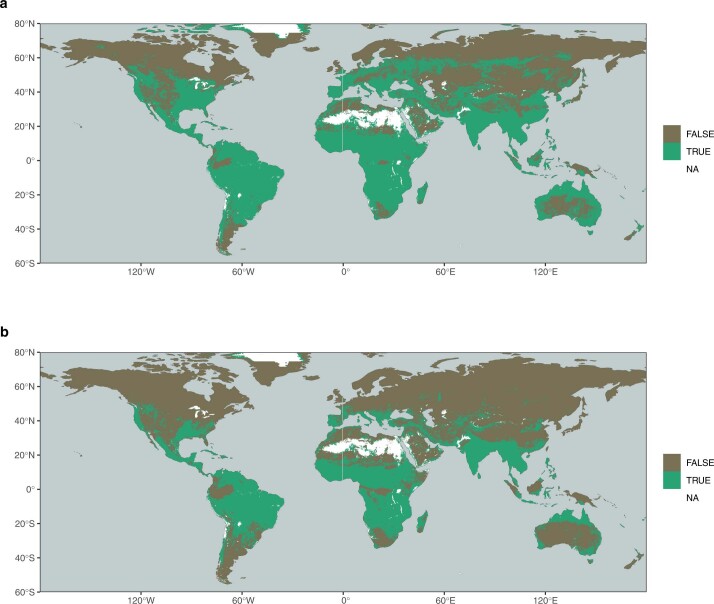
Locations of plant access to deep water storage. Plant access to deep water storage. Green colors indicate regions where *S*_CWDX80_ suggests a rooting zone water storage capacity larger than the integrated water holding capacity across the top 1 m (a) and 2 m (b).

Figure 3a shows the global distribution of *S*_CWDX80_ and reveals patterns across multiple scales—in close agreement with *S*_dSIF_ and *S*_dEF_ (*R*^2^ = 0.76 and *R*^2^ = 0.83, respectively; Supplementary Fig. 3). This indicates that the sensitivity of vegetation activity to an increasing CWD (measured by *S*_dSIF_ and *S*_dEF_) is strongly controlled by hydroclimate (as measured by *S*_CWDX80_). The agreement between *S*_0_ estimates based on water mass-balance approaches^2,26^ and vegetation activity suggests that plants tend to size their roots no deeper, and *S*_0_ no larger, than what is suggested by observed CWD extremes. Magnitudes of *S*_CWDX80_ inferred for 55% (37%) of Earth’s vegetated regions indicate plant access to water stored beyond 1 (2) m soil, assuming texture-dependent WHC^31–33^ (Extended Data Figs. 5 and 6).

**Extended Data Fig. 7:**
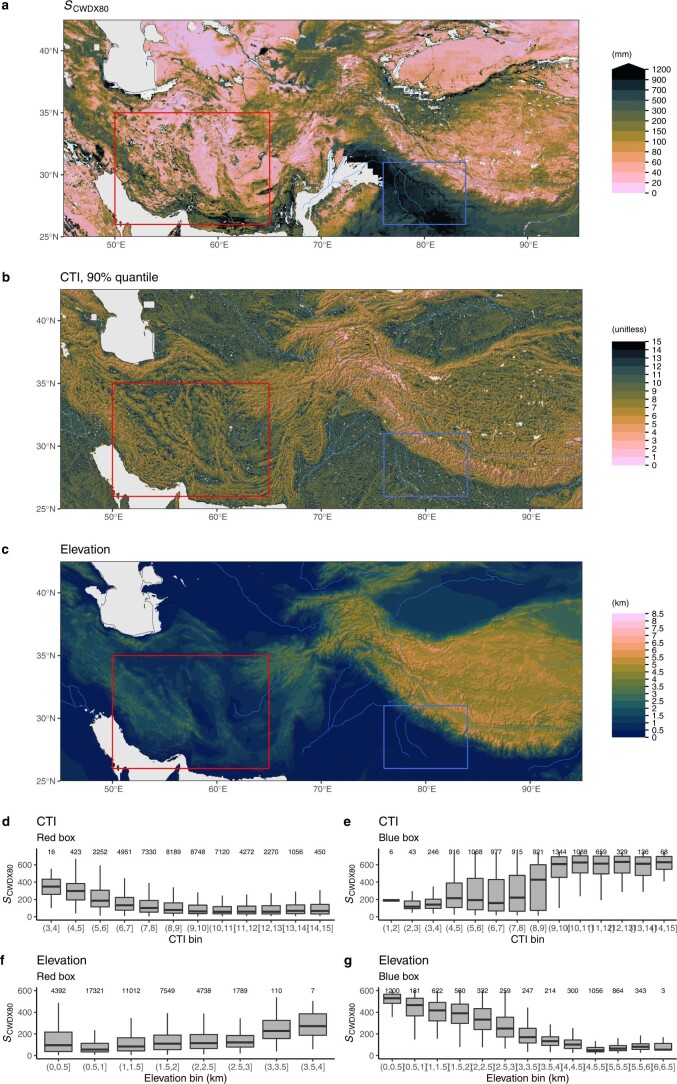
Rooting zone water storage capacity along topographic gradients in central Asia. (a) Rooting zone water storage capacity in central Asia, estimated by the magnitude of cumulative water deficit extreme events with a return period of 80 years *S*_CWDX80_). (b) Compound Topography Index^60^, shown as 90% quantiles of underlying pixels, given at 15 arcsec, within matching 0.05^∘^ gridcells. (c) Elevation from ETOPO1^43^. Red and blue rectangles indicate the domains for which *S*_CWDX80_ distributions along a CTI and an elevation gradient are shown in (d), (e), (f) and (g). The Compound Topography Index (CTI) is a measure for subsurface flow convergence and the water table depth based on the topographical setting^61^. Boxes represent the interquartile ranges of binned values (*Q*_25_, *Q*_75_), and whiskers cover *Q*_25_ − 1.5(*Q*_75_ − *Q*_25_) to *Q*_75_ + 1.5(*Q*_75_ − *Q*_25_). Numbers of data points per bin are given above boxes.

**Extended Data Fig. 8:**
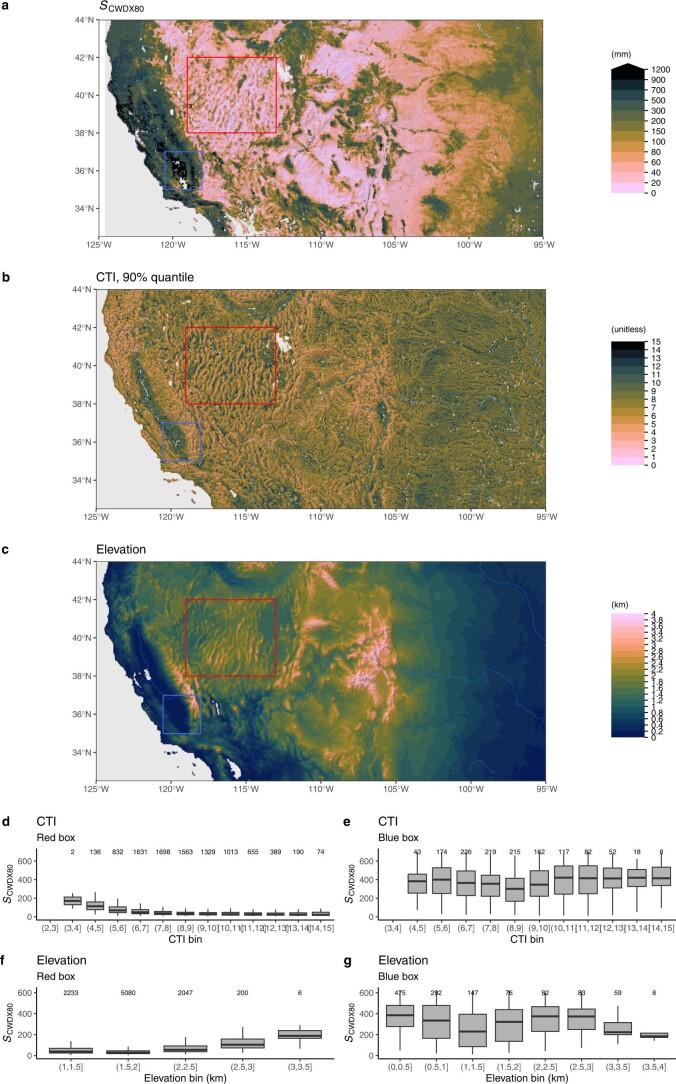
Rooting zone water storage capacity along topographic gradients in the western United States. (a) Rooting zone water storage capacity in the western United States, estimated by the magnitude of cumulative water deficit extreme events with a return period of 80 years *S*_CWDX80_). (b) Compound Topography Index^60^, shown as 90% quantiles of underlying pixels, given at 15 arcsec, within matching 0.05^∘^ gridcells. (c) Elevation from ETOPO1^43^. Red and blue rectangles indicate the domains for which *S*_CWDX80_ distributions along a CTI and an elevation gradient are shown in (d), (e), (f), and (g). The Compound Topography Index (CTI) is a measure for subsurface flow convergence and the water table depth based on the topographical setting^61^. Boxes represent the interquartile ranges of binned values (*Q*_25_, *Q*_75_), and whiskers cover *Q*_25_ − 1.5(*Q*_75_ − *Q*_25_) to *Q*_75_ + 1.5(*Q*_75_ − *Q*_25_). Numbers of data points per bin are given above boxes.

Fine granularity and large spatial heterogeneity of *S*_CWDX80_ at regional scales reveal the importance of land use and the local topographical setting for determining plant-available water-storage capacities (Extended Data Figs. 7 and 8). Complex patterns emerge. Mountainous areas feature higher *S*_CWDX80_ than their surrounding lowlands. In other regions, lowlands feature some of the highest recorded *S*_CWDX80_. In these regions, irrigated agriculture is widespread (Fig. 2 and Extended Data Fig. 1). Variations are likely to extend to even smaller scales along the hillslope topography^7^ and within individual forest stands^34^. These scales lie beyond the resolution of the satellite remote-sensing data used here to calculate CWD.

## Evaluation with rooting-depth observations

The *S*_0_ provides an estimate of the effective total plant-available water, independent of assumptions about physical constraint (limited soil depth, shallow bedrock or groundwater) and independent of uncertain soil texture and water-holding capacity (WHC). Due to the absence of direct observational constraints on *S*_0_, we converted *S*_0_ to a corresponding apparent *z*_r_, enabling an evaluation of *S*_0_ estimates against fully independent observations. We focused on comparing biome-level distributions of inferred apparent rooting depth (*z*_CWDX80_) with a dataset^30^ containing 5,524 individual field observations of plant rooting depth from 1,705 globally distributed sites (Supplementary Fig. 4). We thus tested the link between hydroclimate and below-ground vegetation structure across large climatic gradients.

**Fig. 4:**
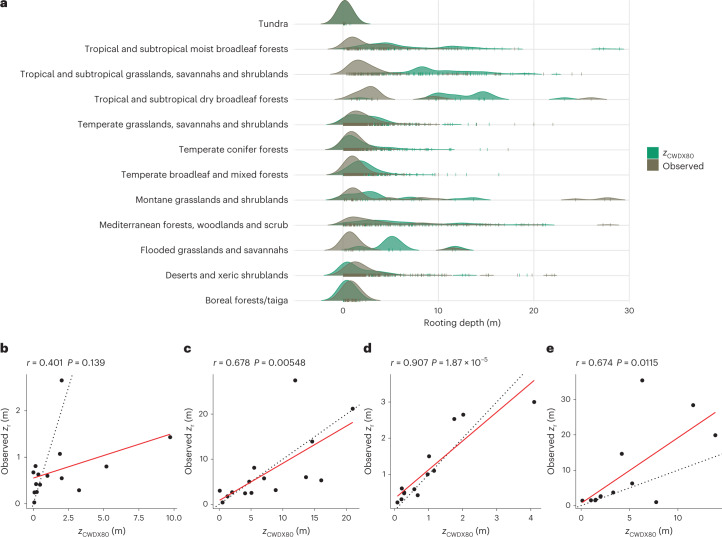
Modelled and observed rooting depth by biome. **a**, Kernel density estimates of observed and predicted (*z*_CWDX80_) rooting depth by biome, based on data aggregated by sites, shown by vertical coloured tick marks. **b**–**e**, The 10% (**b**,**d**) and 90% (**c**,**e**) quantiles of observed versus predicted (*z*_CWDX80_) rooting depth by biome of all data (**b**,**c**) and of a subset of the data where the WTD was measured along with rooting depth (**d**,**e**). Classification of sites into biomes was done on the basis of ref. ^43^. Dotted lines in **b**–**e** represent the 1/1 line. *r* is the Pearson’s correlation coefficient, and *P* is the test statistic based on Pearson’s product moment correlation coefficient.

Predicted and observed biome-level maximum rooting depth (90% quantiles) are correlated (Pearson’s *r* = 0.68; Fig. 4c) while the lower (10%) quantiles appear to be overestimated by *z*_CWDX80_ (Fig. 4b). Using a subset of the data where information about the water-table depth (WTD) is provided (489 entries from 359 sites), we limited values of *z*_CWDX80_ to the value of the observed local WTD (53% of all observations). This yields a strongly improved correlation of observed and estimated biome-level 10% rooting-depth quantiles (Pearson’s *r* = 0.91; Fig. 4d) compared with estimates that are not capped at the observed WTD (Fig. 4b). This suggests that inferred *z*_r_ overestimates values where roots access the groundwater and indicates that groundwater access is relevant across more than half of the globally distributed sites in our dataset. While acting as a constraint on the rooting depth^7^, plant access to groundwater or a perched water table implies sustained transpiration during dry periods, correspondingly large CWDs and, by implication of the model design, large *S*_CWDX80_ and (apparent) *z*_CWDX80_.

## Influence of biotic and abiotic factors

Using first-principles modelling and integrating multiple data streams, we diagnosed a hydrologically effective ecosystem-level *S*_0_ from the sensitivity of vegetation activity to CWD. We found that large-scale variations in *S*_0_ are driven by the hydroclimate and that global patterns of seasonal water deficits are reflected in the rooting depth of plants. More fine-grained variations in *S*_0_ within regions and biomes are linked to land use and irrigation of agricultural land (Fig. 2), to topography (Extended Data Figs. 7 and 8) and to the WTD, as indicated by the comparison with plant-level rooting-depth observations. The method applied here makes use of the sensitivity of remotely sensed ET to an increasing CWD and thus provides estimates of *S*_0_ even if below-ground water stores are never fully depleted during the observational period. Additional analyses, where *S*_0_ was diagnosed from a simple water-balance model with prescribed *S*_0_, confirmed the reliability of the method across a broad range of hydroclimates (Supplementary Text 2 and Supplementary Fig. 5).

The *S*_0_ reflects a combination of biotic and abiotic factors. Biotic factors that determine the total plant-available water are, for example, the rooting depth of the vegetation and plant hydraulic properties. Abiotic factors include the hydroclimate and physical constraints to the rooting depth, related to the texture and depth of the soil and the weathered bedrock^7^. Similarly, human management activities such as irrigation and tile drainage can impact ET, and thus *S*_0_, in agricultural systems. Physical constraints to roots are largely unknown across large scales. Our estimation of *S*_0_ makes no assumptions about such constraints. Instead, the magnitude of the water-storage capacity is inferred from mass-balance considerations. The CWD we derive from the balances of ET and *P* imply that the corresponding amount of water is supplied by local storage or supplied from lateral subsurface water convergence—likely a smaller contributor at the ~5 km spatial resolution of the data analysed here^35^.

Diagnosed values of *S*_0_ implicitly include water intercepted by leaf and branch surfaces, internal plant water storage and moisture stored in the topsoil and supplied to soil evaporation. These components are generally smaller in magnitude compared with moisture storage supplied to transpiration^36^, and their contribution to ET declines rapidly as CWD increases. Hence, spatial variations in *S*_0_ reflect primarily variations mediated by moisture stored across the root zone.

Particularly in regions with pronounced dry seasons, our estimates of *S*_0_ greatly exceed typical values of the total soil WHC when considering the top 1 or 2 m of the soil column and texture information from global databases^31^ (Extended Data Fig. 5). The discrepancy in magnitude and spatial patterns of total 1 (2) m soil WHC and *S*_0_ diagnosed here hints at a critical role of plant access to deep water and the need to extend the focus beyond moisture in the top 1–2 m of soil for understanding and simulating land–atmosphere exchange^10,11^. Indications of widespread plant access to deep water stores are consistent with observations of bedrock-penetrating roots^7,37^ and with evidence for dry-season moisture withdrawal from the weathered bedrock^9,11^. We note that using the global map of *S*_CWDX80_ (*z*_CWDX80_) for directly parameterizing *S*_0_ (*z*_r_) in models may be misleading in areas with particularly small maximum CWDs and consequently small *S*_CWDX80_. Scaling relationships of above- and below-ground plant architecture^30^ and additional effects of how *z*_r_ determines access to below-ground resources and function (for example, nutrients and mechanical stability) should be considered.

Underlying the estimates of *S*_CWDX80_ is the assumption that plant rooting strategies are reflected by CWD extremes with a return period *T* = 80 yr; *S*_dSIF_ and *S*_dEF_ provide an independent constraint to test this assumption. Extended Data Fig. 4 suggests that *T* is not a global constant. A tendency towards higher *T* emerges with an increasing grid-cell average forest-cover fraction.

Our analysis identified mountain regions as being characterized by particularly high *S*_0_, despite shallow soil and regolith depths^38^. This could be due to hillslope-scale variations in groundwater depth, enabling sustained transpiration during prolonged rain-free periods. Lateral subsurface flow at scales beyond the resolution of the data used here (~5 km) may additionally supply water for ET and thus contribute to large inferred *S*_0_ in valley bottoms of large drainage basins. Local convergence (divergence) acts to supply (remove) subsurface moisture and sustain (reduce) ET, leading to larger (smaller) CWD values. Without relying on a priori assumptions regarding *S*_0_ or functional dependencies of water stress effect on ET, thermal infrared- (TIR-) based remote-sensing data (as used here) offer an opportunity to detect such effects^8^. Our analysis yielded strong contrasts in diagnosed *S*_0_ along topographic gradients (Extended Data Figs. 7 and 8). However, further research should assess the accuracy of spatial variations in annual mean ET and potential effects of terrain, where land surface temperature signals on shaded slopes may be misinterpreted by the ALEXI algorithm as signatures of higher ET.

Our global *S*_0_ estimates are a ‘snapshot’ in time. Regional- to continental-scale variations in average tree ages may be associated with changes in rooting depth and *S*_0_. Furthermore, environmental change may trigger changes in vegetation composition and structure^39^, with consequences for *S*_0_. Similarly, deforestation implies changes in rooting depth^18^, *S*_0_ and the surface energy balance^14^. Such temporal changes are not considered here due to the limited length of available time series of satellite observations (16 yr). It remains to be seen whether plasticity in *z*_r_ is sufficiently rapid to keep pace with a changing climate with strong and widespread increases in rainfall variability^40^ and to what degree rising CO_2_ alters plant water use and their carbon economy and thereby the costs and benefits of deep roots.

Taken together, constraints available from local *z*_r_ observations and from global remote sensing of vegetation activity reveal consistent patterns across multiple spatial scales and suggest widespread plant access to deep water storage, including the weathered bedrock and groundwater, or to other ancillary sources of water, such as irrigation. Our study revealed a tight link of the climatology of water deficits and vegetation sensitivity to drought stress. We demonstrated how land–atmosphere interactions and the critical zone water-storage capacity are linked with the rooting depth of vegetation and how below-ground vegetation structure is influenced by the hydroclimate and topography across the globe.

## Methods

### Estimating ET

Unbiased estimates of ET during rain-free periods are essential for determining CWD and estimating *S*_0_ and implied *z*_r_. We tested different remote-sensing-based ET products and found that the ALEXI-TIR product, which is based on TIR remote sensing^28,29^, exhibits no systematic bias during progressing droughts (Supplementary Text 3 and Supplementary Fig. 6), in contrast to other ET estimates assessed here. The stability in ET estimates from ALEXI-TIR during drought is enabled by its effective use of information about the surface energy partitioning, allowing inference of ET rates without reliance on a priori specified and inherently uncertain surface conductances^44^ or shapes of empirical water stress functions^45^, and without assumptions of rooting depth or effective *S*_0_. ALEXI-TIR is thus well suited for estimating actual ET behaviour during drought without introducing circularity in inferring *S*_0_.

### CWD estimation

The CWD is determined here from the cumulative difference of actual ET and the liquid-water infiltration to the soil (*P*_in_). ET is based on thermal infrared remote sensing, provided by the global ALEXI data product at daily and 0.05° resolution, covering years 2003–2018. Values in energy units of the latent heat flux are converted to mass units accounting for the temperature and air-pressure dependence of the latent heat of vaporization following ref. ^46^. The *P*_in_ is based on daily reanalysis data of *P* in the form of rain and snow from WATCH-WFDEI^47^. A simple snow accumulation and melt model^48^ is applied to account for the effect of snowpack as a temporary water storage that supplies *P*_in_ during spring and early summer. Snow melt is assumed to occur above 1 °C and with a rate of 1 mm d^−1^ °C^−1^. The CWD is derived by applying a running sum of (ET – *P*_in_), initiating on the first day when (ET – *P*_in_) is positive (net water loss from the soil) and terminating the summation after rain has reduced the running sum to zero (Supplementary Fig. 7). This yields a continuous CWD time series of daily values. In general, *P* > ET for annual totals. This implies that the CWD summation is initiated at zero each year. In very rare cases, the CWD accumulates over more than one year, and data were discarded if the accumulation extended over five years (‘runaway CWD’). All *P* and snow melt (*P*_in_) are assumed to contribute to reducing the CWD. This implicitly assumes that no run-off occurs while the CWD is above zero. The period between the start and end of accumulation is referred to as a CWD event. Within each event, co-varying data, used for analysis, are removed after rain has reduced the CWD to below 90% of its maximum value within the same event. This concerns the analysis of SIF and EF (see the following) and avoids effects of relieved water stress by re-wetting topsoil layers before the CWD is fully compensated. The algorithm to determine daily CWD values and events is implemented by the R package *cwd*^49^.

### Diagnosing *S*_0_ from vegetation activity

By employing first principles for the constraint of the rooting-zone water availability on vegetation activity^1^, we developed a method to derive how the sensitivity of these fluxes to water stress relates to *S*_0_ and how this sensitivity can be used to reveal effects of access to extensive deep water stores. Two methodologically independent sources of information on vegetation activity were used: EF (defined as ET divided by net radiation) and SIF (normalized by incident short-wave radiation). SIF is a proxy for ecosystem photosynthesis^50^ and is taken here from a spatially downscaled data product^51^ based on GOME-2 data^52,53^. Since net radiation and short-wave radiation are first-order controls on ET and SIF, respectively, and to avoid effects by seasonally varying radiation inputs, we used EF instead of ET and considered the ecosystem-level fluorescence yield, quantified as SIF divided by short-wave radiation (henceforth referred to as ‘SIF’) for all analyses. The resulting estimates for *S*_0_ are referred to as *S*_dEF_ and *S*_dSIF_, respectively.

The principles for relating vegetation activity to the rooting-zone water availability were considered as follows. As the ecosystem-level CWD increases, both gross primary production (ecosystem-level photosynthesis) and ET are limited by the availability of water to plants. In the following, we refer to gross primary production and ET as a generic ‘vegetation activity’ variable *X*(*t*). This principle can be formulated, in its simplest form, as a model of *X*(*t*) being a linear function of the remaining water stored along the rooting zone *S*(*t*), expressed as a fraction of the total rooting-zone water-storage capacity *S*_0_:

1 $$X(t)={X}_{0}\times S(t)/{S}_{0}$$

Following equation (1), *S*_0_ can be interpreted as the total rooting-zone water-storage capacity, or the depth of a water bucket that supplies moisture for ET. Following ref. ^1^ and with *X*(*t*) representing ET, the temporal dynamics during rain-free periods (where run-off can be neglected) are described by the differential equation

2 $${\mathrm{d}}S/{\mathrm{d}}t=-X(t)\Rightarrow {\mathrm{d}}S/{\mathrm{d}}t=-{X}_{0}\times S(t)/{S}_{0}$$

and solved by an exponential function with a characteristic decay timescale *λ*:

3 $$X(t)={X}_{0}\times \exp (-[t-{t}_{0}]/\lambda )$$

*λ* is related to *S*_0_ as *S*_0_ = *λX*_0_, where *X*_0_ is the initial ET at *S*(*t*_0_) = *S*_0_. In other words, the apparent observed exponential ET decay timescale *λ*, together with *X*_0_, reflects the total rooting-zone water-storage capacity *S*_0_.

Fitting exponentials from observational data is subject to assumptions regarding stomatal responses to declines in *S*(*t*) and is relatively sensitive to data scatter. Hence, resulting estimates of *S*_0_ may not be robust. With CWD(*t*) = *S*_0_ − *S*(*t*) and equation (1), the relationship of *X*(*t*) and CWD(*t*) can be expressed as a linear function

4 $$X(t)={X}_{0}-{X}_{0}/{S}_{0}\times {{{\rm{CWD}}}}(t)$$

and observational data for *X*(*t*) can be used to fit a linear regression model. Its intercept *a* and slope *b* can then be used as an alternative, and potentially more robust, estimate for *S*_0_:

5 $${S}_{0}=-a/b$$

This has the further advantage that estimates for *S*_0_ can be derived using any observable quantity of vegetation activity *X*(*t*) (not just ET as in ref. ^1^) under the assumption that activity attains zero at the point when the CWD reaches the total rooting-zone water-storage capacity; that is, *X*(t^*^) = 0 for CWD(t^*^) = *S*_0_.

Here we use a spatially downscaled product of SIF^51^, normalized by incident short-wave radiation (WATCH-WFDEI data^47^), and the EF, defined as the ratio of ET (ALEXI-ET data^29^) over net radiation (GLASS data^54^), as two alternative, normalized proxies for water-constrained vegetation activity, termed ${X}^{{\prime} }$. Normalization by net radiation and incident short-wave radiation, respectively, removes effects by seasonally varying energy available for vegetation activity. ${X}_{0}^{{\prime} }$ is thus assumed to be stationary over time, and the relationship of ${X}^{{\prime} }(t)$ and CWD(*t*) is interpreted here as a reflection of effects by below-ground water availability and used to derive *S*_dSIF_ and *S*_dEF_. All data used for ${X}_{0}^{{\prime} }$ are provided at 0.05° and daily resolution.

*S*_dSIF_ and *S*_dEF_ were then derived on the basis of the relationship of EF and normalized SIF versus CWD, guided by equation (5). The relationship was analysed for each pixel with pooled data belonging to the single largest CWD event of each year and using the 90% quantile of EF and normalized SIF within 50 evenly spaced bins along the CWD axis. Binning and considering percentiles were chosen to reduce effects of vegetation activity reduction due to factors other than water stress (CWD). We then tested, for each pixel, whether the data support the model of a single linear decline of SIF (EF) with increasing CWD (equation (5)) or, alternatively, a segmented regression model with one or two change points, using the R package *segmented*^55^. The model with the lowest Bayesian information criterion was chosen, and *S*_dSIF_ and *S*_dEF_ were quantified only for pixels where no significant change point was detected and where the regression of EF (SIF) versus CWD had a significantly negative slope. Flattening EF (SIF) versus CWD relationships were identified where a significant change point was detected and where the slope of the second regression segment was significantly less negative (*P* = 0.05 of *t* test) compared with the slope of the first segment. Examples, visualizing the diagnosing of *S*_0_ from EF, are given in Supplementary Fig. 8. We performed additional tests of the method’s reliability in estimating *S*_0_ by deriving *S*_dEF_ from simulations of the ecosystem water balance and ET, where *S*_0_ was prescribed, using the SPLASH (Simple Process-Led Algorithms for Simulating Habitats) model^46^. This demonstrates that the method applied for *S*_dSIF_ and *S*_dEF_ yields accurate estimates of *S*_0_ across all climatic conditions and independent of the size of *S*_0_ (Supplementary Text 2 and Supplementary Fig. 5).

### Diagnosing *S*_0_ from CWDs

Following ref. ^2^, the *S*_0_ is estimated on the basis of CWD extremes occurring with a return period of *T* years. Magnitudes of extremes with a given return period *T* (*S*_CWDX*T*_) are estimated by fitting an extreme value distribution (Gumbel) to the annual maximum CWD values for each pixel separately, using the *extRemes* R package^56^. Values *S*_CWDX*T*_. are translated into an effective depth *z*_CWDX*T*_ using estimates of the plant-available soil WHC, on the basis of soil-texture data from a gridded version of the Harmonized World Soil Database^31,32^ and pedo-transfer functions derived by ref. ^33^. Associations of *S*_CWDX*T*_ and topography were analysed considering the Compound Topography Index^57^ and elevation from ETOPO1^58^. The Compound Topography Index is a measure for subsurface flow convergence and the WTB based on the topographical setting^59^.

### Estimating return periods

Diagnosed values of *S*_dSIF_ and *S*_dEF_ provide a constraint on the return period *T*. To yield stable estimates of *T* and avoid effects of the strong nonlinearity of the function to derive *T* from the fitted extreme value distributions and magnitudes estimated by *S*_dSIF_ and *S*_dEF_, we pooled estimates *S*_dSIF_ (*S*_dEF_) and *S*_CWDX*T*_ values within 1° pixels (≤400 values). A range of discrete values *T* was screened (10, 20, 30, 40, 50, 60, 70, 80, 90, 100, 150, 200, 250, 300, 350, 400, 450, 500 yr), and the best estimate *T* was chosen on the basis of comparison with *S*_dSIF_ (*T*_SIF_) and to *S*_dEF_ (*T*_EF_), that is, where the absolute value of the median of the logarithm of the bias was minimal. Relationships of best matching *T* with topography (measured by the Compound Topography Index^57^) and with the forest-cover fraction (MODIS MOD44B^60^) were analysed.

### Rooting-depth estimation and observations

We converted root-zone water-storage capacity estimates, *S*_CWDX80_, to a corresponding apparent rooting depth (*z*_CWDX80_) using a global soil-texture map^31,32^. The conversion of *S*_CWDX80_ into a corresponding depth *z*_CWDX80_ accounts for topsoil and subsoil texture and WHC along the rooting profile (Methods and Fig. 3b) and, in view of lacking information with global coverage about the WHC of the weathered bedrock, assuming uniform subsoil texture extending below 30 cm depth. The comparison of biome-level quantities (instead of a direct point-by-point comparison) avoids the inevitable scale mismatch between in situ plant-level observations and global remote-sensing data.

The observational rooting-depth dataset (*N* = 5,524) was compiled by ref. ^30^ by combining and complementing published datasets from refs. ^22,7^. The data include observations of the maximum rooting depth of plants taken from 361 published studies plus additional environmental and climate data. The *z*_r_ was taken as the plant’s maximum rooting depth. Data were aggregated by sites (*N* = 1,705) on the basis of longitude and latitude information. Sites were classified into biomes using maps of terrestrial ecoregions^43^. Quantiles (10%, 90%) were determined for each biome. For a subset of the data (359 sites) where parallel measurements of the WTD were available, we conducted the same analysis but took the minimum of WTD and *z*_r_.

## Online content

Any methods, additional references, Nature Portfolio reporting summaries, source data, extended data, supplementary information, acknowledgements, peer review information; details of author contributions and competing interests; and statements of data and code availability are available at 10.1038/s41561-023-01125-2.

## Supplementary information

Supplementary Information Supplementary Figs. 1–8 and Texts 1–3.

## Data Availability

Global datasets of *S*_CWDX80_ and *z*_CWDX80_ are available on *Zenodo*^61^. The rooting-depth data are published separately^30^.

## Code availability

The CWD calculation from ET and *P*_in_ time series is implemented by the R package *cwd*^49^. All code for this analysis is published on *Zenodo*^62^.
