# Supplementary material for: Global patterns of water storage in the rooting zones of vegetation
Source: Nat Geosci. 2023 Feb 9;16(3):250–6. doi: 10.1038/s41561-023-01125-2 (PMC10005945; doi:10.1038/s41561-023-01125-2)
Supplement: Supplementary file 1 — Supplementary Figs. 1–8 and Texts 1–3. [file 41561_2023_1125_MOESM1_ESM.pdf]

nature geoscience

Article

https://doi.org/10.1038/s41561-023-01125-2

Global patterns of water storage in the rooting zones of vegetation

In the format provided by the authors and unedited

SUPPORTING INFORMATION
Global patterns of water storage in the rooting zones of vegetation

Benjamin D. Stocker, Shersingh Joseph Tumber-Dávila, Alexandra Konings, Martha C. Anderson, Christopher Hain, Robert B. Jackson

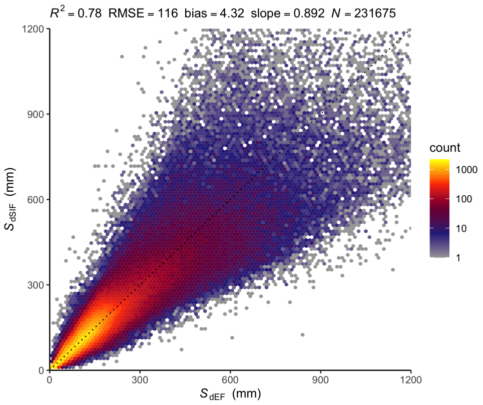

Figure S1: Correlation of S_0, diagnosed from EF (S_{dEF}) and from SIF (S_{dSIF}). Metrics of the correlation are given by text annotation on top of the figures (RMSE is the root mean square error). The dotted black line indicates the 1:1 line.

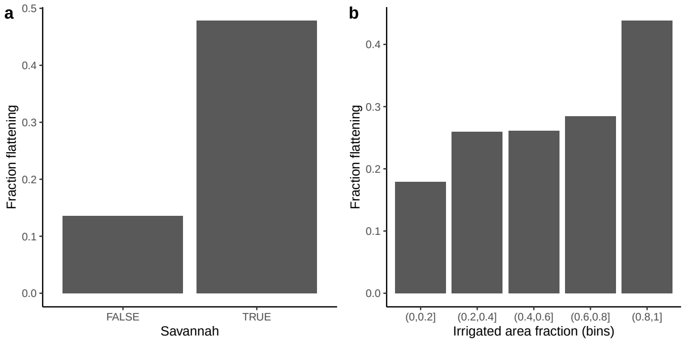

Figure S2: Fraction of grid cells where a flattening relationship between EF and CWD was detected (see Methods), in savannahs and outside (a) and depending on the irrigated area fraction (b). 'Savannah' is taken as 'Woody Savannah' from MODIS MCD12C1 for year 2010 Friedl and Sulla-Menashe (2015). The fraction of irrigated areas is from Siebert et al. (2005), taken as actually irrigated areas as a fraction of land area.

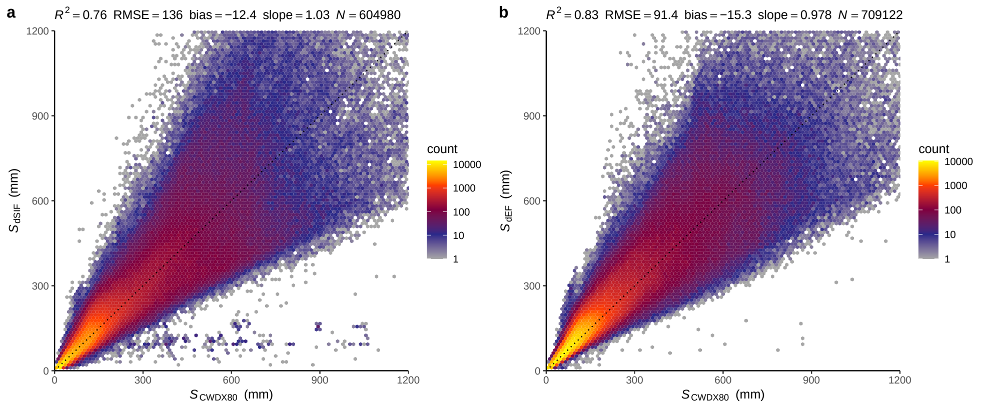

Figure S3: Correlation of S_{dEF} (a) and S_{dSIF} (b) with S_{CWDX80}. Metrics of the correlation are given by text annotation on top of the figures (RMSE is the root mean square error). The dashed black line indicates the 1:1 line.

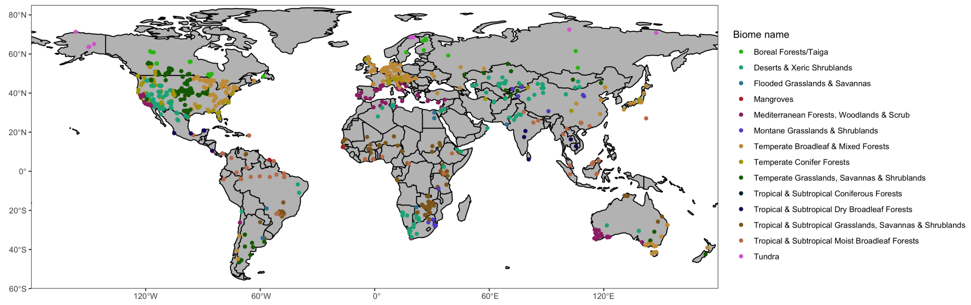

Figure S4: Location and biome type of sites where rooting depth data is provided and used here for comparison against z_{CWDX80}. Biome classification was done based on Olson et al. (2001).

S1 Estimating return periods

Underlying the estimates of S_{CWDX80} is the assumption that plant rooting strategies are reflected by CWD extremes with a return period $T = 80$ years. We analysed the sensitivity of our estimates of CWD extremes to different choices of the extreme event return interval T and how the different $S_{\mathrm{CWDX}T}$ compare to S_{dSIF} and S_{dEF}. As described in the main text, we found a clear tendency towards higher T emerges with an increasing gridcell average forest cover fraction (Extended Data Fig. 4d) and a tendency, albeit a weaker one, towards higher T in areas of subsurface water convergence (measured by a high Compound Topograhy index, CTI).

In regions with a high forest cover fraction, S_0 appears to be adapted to 500-year and even rarer CWD events, while other regions are associated with lower values of T. In other words, vegetation activity declines when plants experience a CWD extreme that occurs with an expected return period, related to the life form of dominant plants. S_0 appears to be adapted to 500-year and even rarer events in regions with a high forest cover fraction, but to lower T outside. A return period on the order of multiple centuries is consistent with the typical lifetime of trees Brienen et al. (2020) and suggests that optimal plant adaptation of life history strategies to a stochastic environment may be understood as being governed by frequencies of climate extremes in relation to the lifetime of affected organisms. Interestingly, substantial variations in T remain even within land cover types (e.g., the boreal forests of Russia). This variation may be related to the large-scale topographical setting and the tendency towards shallow groundwater table depths, as measured by the CTI (Marthews et al. (2015), Extended Data Fig. 4c). In such areas, vegetation appears to sustain particularly rare CWD extremes (diagnosed here from its low sensitivity to CWD during the observation period), possibly enabled by roots' access to a relatively shallow saturated zone.

S2 Testing the method for diagnosing S_0

To test the ability of our method to reliably diagnose the true S_0 from EF and the SIF, normalised by the shortwave radiation, we performed model simulations and "reverse engineered" S_0, prescribed to the model. For this, we used SPLASH Davis et al. (2017), a relatively simple model for the surface water and energy exchanges across the globe, requiring minimal inputs to be specified, and implemented as part of the *rsofun* R package Stocker et al. (2021). The model simulates potential ET (PET) as a function of net radiation and the energy demand for vaporization based on the Priestly-Taylor Priestley and Taylor (1972). Actual ET (AET) is simulated as the minimum of the "demand" from PET and the "supply", which is a function of water availability across the rooting zone. The water balance is simulated with infiltration of liquid water given by precipitation plus snow melt, and runoff is generated when a single rooting zone water storage capacity is saturated. This corresponds to a "bucket-model" approach and relies on the depth of the bucket, reflecting S_0, to be specified *a priori*.

Three sets of point-scale model simulations were performed with forcings reflecting the climate at the locations (N=1705) where rooting depth observations were given as used in the main text of the present study. In the first two sets of simulations, S_0 (bucket depth) was prescribed to 100 and 200 mm, respectively. In the third set of simulations, S_0 was prescribed to the value diagnosed from S_{dEF}. Together, this allows us to test how accurate the method is in diagnosing S_0 from the relationship of the evaporative fraction (EF) and the cumulative water deficit (CWD), and to evaluate whether the reliability of the method is subject to the hydroclimate at different sites and thus to the magnitude of water deficits. Diagnosing the real effective S_0 from remote sensing observations is additionally subject to uncertainty in estimates of ET, P, etc. Diagnosing it from the model output allows us to test the method without confounding effects by uncertain data. However, it should be noted that simulated relationships are idealized and thus likely simpler to diagnose by our method than real relationships.

Fig. S5 demonstrates that our method for diagnosing S_0 yields accurate estimates of the (prescribed) true S_0. The accuracy is high both under a setup where the true S_0 is relatively small (100 mm) or large (200, see Fig. S5a), and performs reliably across a large range of prescribed S_0 and climates, capturing 88% of the prescribed (true) variation in S_0 across globally distributed sites (Fig. S5b). The method yields accurate estimates also under conditions where the true S_0 is large in comparison to typical annual maximum CWD (see, e.g., 'Tundra', 'Boreal Forests/Taiga', or 'Tropical Subtropical Moist Forests for simulations with prescribed S_0 of 200 mm in Fig. S5c).

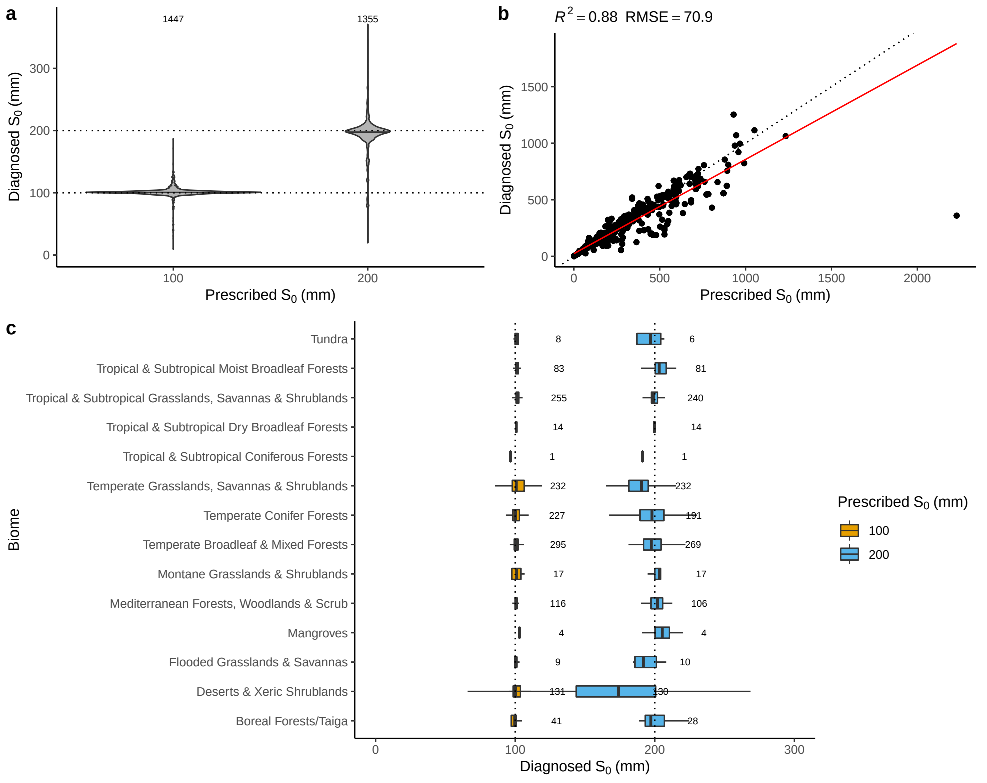

Figure S5: Method evaluation for diagnosing S_0 for three sets of site-level simulations, where S_0 was prescribed to 100 mm and 200 mm (a, c), and to the value diagnosed based on the evaporative fraction (S_{dEF}) at site locations for which rooting depth observations were used in the present study (b). The distrribution of values is given in (a) by violin plots. Numbers of data points per bin are given above violin plots. The dotted black line in (b) indicates the 1:1 line. Boxes in (c) represent the interquartile ranges of the values (Q_{25}, Q_{75}), and whiskers cover $Q_{25} - 1.5(Q_{75} - Q_{25})$ to $Q_{75} + 1.5(Q_{75} - Q_{25})$. Numbers of data points per bin are given next to boxes.

S3 Evaluating ET datasets

Unbiased estimates of ET during rain-free periods are essential for implying rooting depth following the method employed here. Various global ET data products are available. The widely-used Priestly-Taylor (PT) model uses net radiation for estimating the energy supply available for evapotranspiration. Additional empirical scalars have to be applied to reduce ET estimates under water-limited conditions. The Penman-Monteith (PM) model is conceptually related to the PT model, but explicitly resolves effects of atmospheric dryness (vapour pressure deficit) and multiple conductance terms that control ET. As for the PT model, water stress effects have to be factored in additionally, commonly by reducing the surface conductance to transpiration.

The challenge with both PT and PM models is that water stress factors themselves rely, either directly or indirectly, on assumptions regarding plant rooting depth and are thus not suitable for use with the methods applied here. They would introduce circular reasoning and the implied rooting depth would directly reflect the assumptions regarding rooting depth or sensitivity of conductance to water stress, introduced in the PT and PM models themselves. Moreover, if rooting depth assumptions (and thus assumptions of effective S_0) are inaccurate, respective ET estimates should exhibit a systematic bias related to the severity of water stress. A potential solution to this problem is to rely on ET products that make no a priori assumptions regarding rooting depth. Thermal infrared (TIR) -based methods rely primarily on land surface and air properties for estimating ET. However, previous studies found generally larger scatter in TIR-based ET estimates. For the present analysis, it's particularly important that estimates exhibit no systematic bias and that the bias is not related to the duration of rain-free periods and the severity of water stress. Therefore, we first tested a set of ET-based datasets with global coverage, before using the data for the present analysis.

We evaluated the bias of modelled versus observed ET, measured with the eddy covariance technique. The data were provided through the FLUXNET 2015 Tier 1 dataset Pastorello et al. (2021). To focus the evaluation on model performance under water stress, we subset the data to sites and periods where clear effects of water stress on photosynthetic light use efficiency have been identified Stocker et al. (2018). Data were aligned by the onset of periods with apparent water stress effects (droughts) to determine the "day into drought" (*dday*). Data were then normalised to pre-drought levels, separately for each site, and aggregated across drought events and years. Finally, we calculated quantiles of the normalised, aggregated bias versus *dday*.

Fig. S6 reveals that indeed, although exhibiting less scatter before, the PT and PM-based algorithms perform less reliably than the TIR-based algorithm during rain-free periods. ALEXI-TIR provides accurate estimates of ET with no systematic bias related to the duration of droughts and the severity of water stress, and provides a robust observation of surface water loss. Since ALEXI-TIR makes no assumptions regarding rooting depth or effective S_0, and in view of its robust performance under water-stressed conditions, we apply this data product for all analyses shown here.

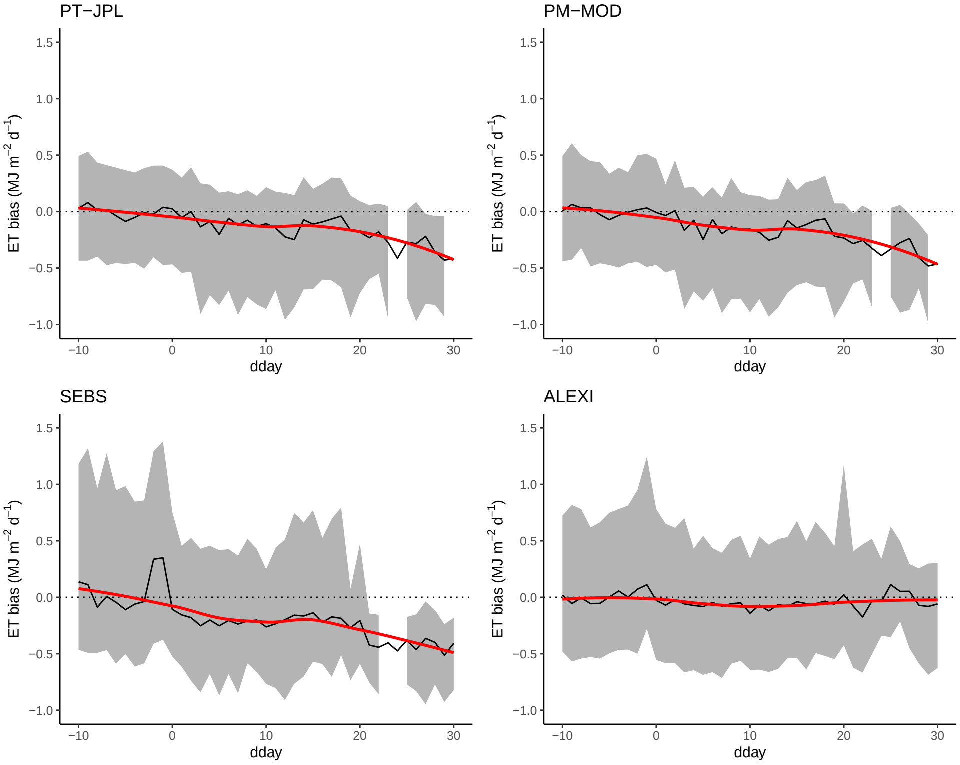

Figure S6: Bias of modelled versus observed ET based on four different algorithms applied to remote sensing data, and evaluated with observations from ecosystem flux measurements (FLUXNET Eddy covariance) collected during periods of drought. The black lines indicate the median bias for each day into the drought ('dday'), derived from multiple drought events and sites. The shaded area expands from the 33 to the 66 percentile, the red line is a LOESS smoothing line based on the median.

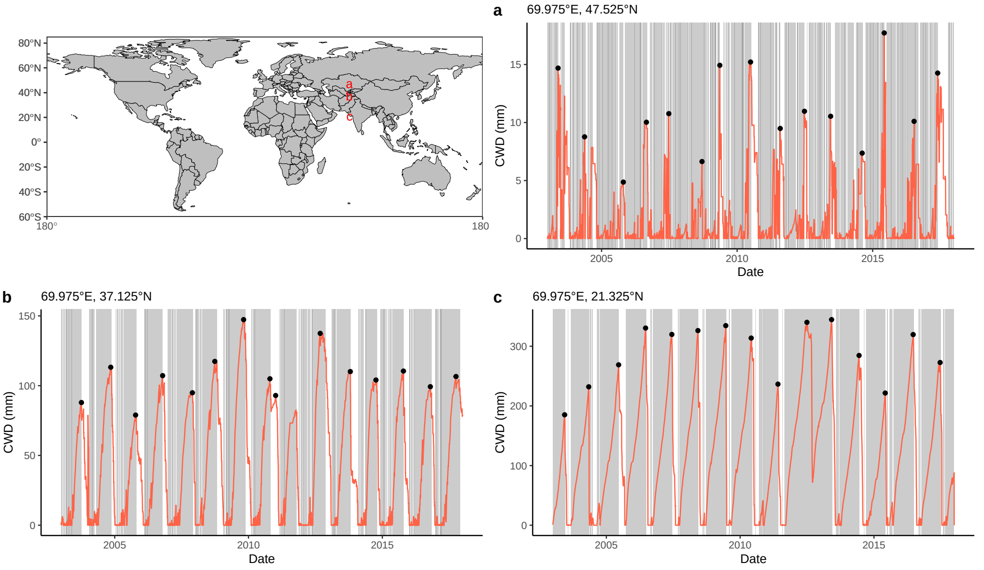

Figure S7: Cumulative water deficit (CWD) time series for three example gridcells (a, b, c; indicated on the map). Time series of CWD are given by the red line. Black dots indicate maximum CWD values for each calendar year, as used for the extreme value analysis and estimating magnitudes of extreme events. The grey bands indicate periods of continuously increasing CWD, referred to as CWD *events*. Panel (c) shows an example in which a CWD is not fully compensated by the wet season in year 2012.

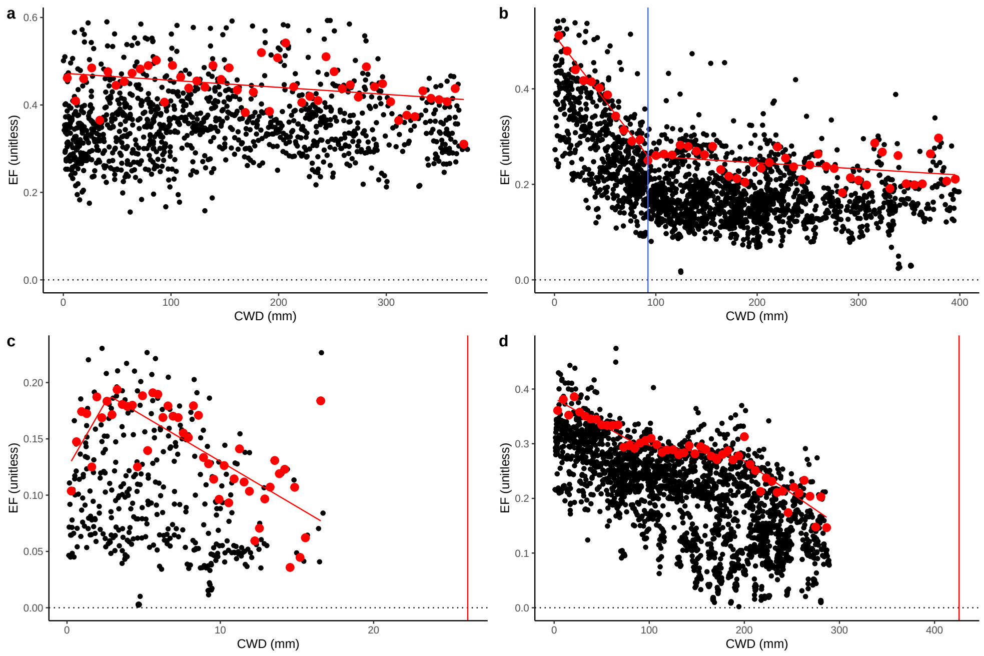

Figure S8: Visualisation of method to infer S_0 from the relationship of the evaporative fraction (EF) and the cumulative water deficit (CWD), yielding S_{dEF}. Examples are given for four gridcells, where S_{dEF} could not be determined (a, site at -95.0833°E, 18.5833°N), where a flattening relationship was detected around a CWD as illustrated by the vertical blue line (b, site at 117.176°E, -32.9998°N), and where S_{dEF} was determined (c, site at -106.6469°E, 35.3985°N; d, site at -120.9669°E, 38.4314°N) as illustrated by the vertical red line.

References

Brienen, R. J. W., Caldwell, L., Duchesne, L., Voelker, S., Barichivich, J., Baliva, M., Ceccantini, G., Di Filippo, A., Helama, S., Locosselli, G. M., Lopez, L., Piovesan, G., Schöngart, J., Villalba, R., and Gloor, E.: Forest carbon sink neutralized by pervasive growth-lifespan trade-offs, Nat. Commun., 11, 4241, 2020.

Davis, T. W., Prentice, I. C., Stocker, B. D., Thomas, R. T., Whitley, R. J., Wang, H., Evans, B. J., Gallego-Sala, A. V., Sykes, M. T., and Cramer, W.: Simple process-led algorithms for simulating habitats (SPLASH v.1.0): robust indices of radiation, evapotranspiration and plant-available moisture, Geoscientific Model Development, 10, 689–708, 2017.

Friedl, M. and Sulla-Menashe, D.: MCD12C1 MODIS/Terra+Aqua Land Cover Type Yearly L3 Global 0.05Deg CMG V006, 2015.

Marthews, T. R., Dadson, S. J., Lehner, B., Abele, S., and Gedney, N.: High-resolution global topographic index values for use in large-scale hydrological modelling, Hydrology and Earth System Sciences, 19, 91–104, https://doi.org/10.5194/hess-19-91-2015, URL `https://hess.copernicus.org/articles/19/91/2015/`, 2015.

Olson, D. M., Dinerstein, E., Wikramanayake, E. D., Burgess, N. D., Powell, G. V. N., Underwood, E. C., D'amico, J. A., Itoua, I., Strand, H. E., Morrison, J. C., Loucks, C. J., Allnutt, T. F., Ricketts, T. H., Kura, Y., Lamoreux, J. F., Wettengel, W. W., Hedao, P., and Kassem, K. R.: Terrestrial Ecoregions of the World: A New Map of Life on Earth, BioScience, 51, 933, 2001.

Pastorello, G., Trotta, C., Canfora, E., Chu, H., Christianson, D., Cheah, Y.-W., Poindexter, C., Chen, J., Elbashandy, A., Humphrey, M., Isaac, P., Polidori, D., Reichstein, M., Ribeca, A., van Ingen, C., Vuichard, N., Zhang, L., Amiro, B., Ammann, C., Arain, M. A., Ardö, J., Arkebauer, T., Arndt, S. K., Arriga, N., Aubinet, M., Aurela, M., Baldocchi, D., Barr, A., Beamesderfer, E., Marchesini, L. B., Bergeron, O., Beringer, J., Bernhofer, C., Berveiller, D., Billesbach, D., Black, T. A., Blanken, P. D., Bohrer, G., Boike, J., Bolstad, P. V., Bonal, D., Bonnefond, J.-M., Bowling, D. R., Bracho, R., Brodeur, J., Brümmer, C., Buchmann, N., Burban, B., Burns, S. P., Buysse, P., Cale, P., Cavagna, M., Cellier, P., Chen, S., Chini, I., Christensen, T. R., Cleverly, J., Collalti, A., Consalvo, C., Cook, B. D., Cook, D., Coursolle, C., Cremonese, E., Curtis, P. S., D'Andrea, E., da Rocha, H., Dai, X., Davis, K. J., De Cinti, B., de Grandcourt, A., De Ligne, A., De Oliveira, R. C., Delpierre, N., Desai, A. R., Di Bella, C. M., di Tommasi, P., Dolman, H., Domingo, F., Dong, G., Dore, S., Duce, P., Dufrêne, E., Dunn, A., Dušek, J., Eamus, D., Eichelmann, U., ElKhidir, H. A. M., Eugster, W., Ewenz, C. M., Ewers, B., Famulari, D., Fares, S., Feigenwinter, I., Feitz, A., Fensholt, R., Filippa, G., Fischer, M., Frank, J., Galvagno, M., Gharun, M., Gianelle, D., Gielen, B., Gioli, B., Gitelson, A., Goded, I., Goeckede, M., Goldstein, A. H., Gough, C. M., Goulden, M. L., Graf, A., Griebel, A., Gruening, C., Grünwald, T., Hammerle, A., Han, S., Han, X., Hansen, B. U., Hanson, C., Hatakka, J., He, Y., Hehn, M., Heinesch, B., Hinko-Najera, N., Hörtnagl, L., Hutley, L., Ibrom, A., Ikawa, H., Jackowicz-Korczynski, M., Janouš, D., Jans, W., Jassal, R., Jiang, S., Kato, T., Khomik, M., Klatt, J., Knohl, A., Knox, S., Kobayashi, H., Koerber, G., Kolle, O., Kosugi, Y., Kotani,

A., Kowalski, A., Kruijt, B., Kurbatova, J., Kutsch, W. L., Kwon, H., Launiainen, S., Laurila, T., Law, B., Leuning, R., Li, Y., Liddell, M., Limousin, J.-M., Lion, M., Liska, A. J., Lohila, A., López-Ballesteros, A., López-Blanco, E., Loubet, B., Loustau, D., Lucas-Moffat, A., Lüers, J., Ma, S., Macfarlane, C., Magliulo, V., Maier, R., Mammarella, I., Manca, G., Marcolla, B., Margolis, H. A., Marras, S., Massman, W., Mastepanov, M., Matamala, R., Matthes, J. H., Mazzenga, F., McCaughey, H., McHugh, I., McMillan, A. M. S., Merbold, L., Meyer, W., Meyers, T., Miller, S. D., Minerbi, S., Moderow, U., Monson, R. K., Montagnani, L., Moore, C. E., Moors, E., Moreaux, V., Moureaux, C., Munger, J. W., Nakai, T., Neirynck, J., Nesic, Z., Nicolini, G., Noormets, A., Northwood, M., Nosetto, M., Nouvellon, Y., Novick, K., Oechel, W., Olesen, J. E., Ourcival, J.-M., Papuga, S. A., Parmentier, F.-J., Paul-Limoges, E., Pavelka, M., Peichl, M., Pendall, E., Phillips, R. P., Pilegaard, K., Pirk, N., Posse, G., Powell, T., Prasse, H., Prober, S. M., Rambal, S., Rannik, Ü., Raz-Yaseef, N., Rebmann, C., Reed, D., de Dios, V. R., Restrepo-Coupe, N., Reverter, B. R., Roland, M., Sabbatini, S., Sachs, T., Saleska, S. R., Sánchez-Cañete, E. P., Sanchez-Mejia, Z. M., Schmid, H. P., Schmidt, M., Schneider, K., Schrader, F., Schroder, I., Scott, R. L., Sedlák, P., Serrano-Ortíz, P., Shao, C., Shi, P., Shironya, I., Siebicke, L., Šigut, L., Silberstein, R., Sirca, C., Spano, D., Steinbrecher, R., Stevens, R. M., Sturtevant, C., Suyker, A., Tagesson, T., Takanashi, S., Tang, Y., Tapper, N., Thom, J., Tomassucci, M., Tuovinen, J.-P., Urbanski, S., Valentini, R., van der Molen, M., van Gorsel, E., van Huissteden, K., Varlagin, A., Verfaillie, J., Vesala, T., Vincke, C., Vitale, D., Vygodskaya, N., Walker, J. P., Walter-Shea, E., Wang, H., Weber, R., Westermann, S., Wille, C., Wofsy, S., Wohlfahrt, G., Wolf, S., Woodgate, W., Li, Y., Zampedri, R., Zhang, J., Zhou, G., Zona, D., Agarwal, D., Biraud, S., Torn, M., and Papale, D.: Author Correction: The FLUXNET2015 dataset and the ONEFlux processing pipeline for eddy covariance data, Sci Data, 8, 72, 2021.

Priestley, C. H. B. and Taylor, R. J.: On the assessment of surface heat flux and evaporation using large-scale parameters, Monthly Weather Review, 100, 81–92, 1972.

Siebert, S., Döll, P., Hoogeveen, J., Faures, J.-M., Frenken, K., and Feick, S.: Development and validation of the global map of irrigation areas, Hydrology and Earth System Sciences, 9, 535–547, 2005.

Stocker, B., Marqués, L., and Hufkens, K.: rsofun v4.0 Modelling framework for site-scale simulations of ecosystem processes in R, https://doi.org/10.5281/zenodo.5530824, URL `https://doi.org/10.5281/zenodo.5530824`, 2021.

Stocker, B. D., Zscheischler, J., Keenan, T. F., Colin Prentice, I., Peñuelas, J., and Seneviratne, S. I.: Quantifying soil moisture impacts on light use efficiency across biomes, New Phytologist, 218, 1430–1449, 2018.
